# Recent Advances in Conductive Composite Hydrogels for Electronic Skin Applications

**DOI:** 10.3390/gels11100822

**Published:** 2025-10-13

**Authors:** Yiqing Yuan, Yilong Zhang, Haiyang Duan, Yitao Zhang, Lijun Lu, Artem Emel’yanov, Alexander S. Pozdnyakov, Pengcheng Zhu, Yanchao Mao

**Affiliations:** 1 Key Laboratory of Materials Physics of Ministry of Education, School of Physics, Zhengzhou University, Zhengzhou 450001, China; 2 A.E. Favorsky Irkutsk Institute of Chemistry, Siberian Branch of the Russian Academy of Sciences, Irkutsk 664033, Russia; emelyanov@irioch.irk.ru (A.E.); pozdnyakov@irioch.irk.ru (A.S.P.)

**Keywords:** conductive composite hydrogels, electronic skins, conductive fillers, human–machine interaction, electrophysiological monitoring

## Abstract

Electronic skins (E-skins) are the integration of intelligent wearable sensors that can collect human physiological, motion, or environmental parameters in real-time through flexible, sensitive materials. The performance of E-skins depends on the selection of materials to a large extent. Hydrogel materials are an excellent candidate for E-skin preparation due to their tissue-like softness and biocompatibility. However, their low electrical conductivity, weak mechanical strength, and environmental instability seriously hinder high-fidelity signal acquisition and reliable operation in practical applications. To overcome these bottlenecks, conductive composite hydrogels have emerged as a promising alternative material. The unique properties of conductive composite hydrogels, such as high stretchability, self-healing ability, and adjustable electrical conductivity, address the relevant issues of traditional hydrogels in wearable applications. This review focuses on conductive composite hydrogels for wearable E-skins. Firstly, the types, characteristics, and preparation strategies of hydrogel matrix materials are introduced. Subsequently, the performance regulation mechanisms of key conductive fillers on composite hydrogels are discussed. Then, the application progress in electrophysiological signal monitoring, human–machine interaction, and human motion monitoring is reviewed. Finally, the current challenges and future development directions of hydrogel-based E-skins are prospected, aiming to provide comprehensive material and fabrication references for the practical application of composite hydrogel in electronic skins.

## 1. Introduction

In recent years, electronic skins have been developing rapidly in the fields of health monitoring, human–machine interaction (HMI), and motion tracking, gradually becoming a key technology that connects human physiological signals with electronic systems [1,2,3]. As flexible devices, they offer unique advantages over traditional rigid sensors, enabling non-invasive, real-time, and continuous monitoring in scenarios such as clinical diagnosis, daily health management, and intelligent interaction [3,4,5]. However, traditional E-skin sensing devices still face many challenges in practical applications. Firstly, most traditional devices lack sufficient flexibility and stretchability, which cannot synchronize with the deformation of human skin or tissues during movement and are prone to signal distortion when capturing subtle physiological activities such as muscle tremors [6,7]. Secondly, they often face biocompatibility and skin adhesion issues, which require reliance on external fixing methods. Long-term use may cause skin irritation and fail to maintain stable contact with the skin during complex movements [8,9,10]. In addition, there is still much room for improvement in sensing performance. The sensitivity and response time of traditional sensors are difficult to meet the needs of high-precision monitoring, such as distinguishing small changes in joint angles [11,12]. Behind these limitations, material performance is a key influencing factor. Traditional hydrogels have been studied as main fabrication materials, possessing tissue-like softness and biocompatibility, which can reduce skin irritation and discomfort [13]. However, they also have several shortcomings that need to be solved. Low electrical conductivity easily leads to signal loss or distortion. Weak mechanical strength makes them prone to damage during joint motion monitoring (e.g., elbow and knee flexion), and repetitive mechanical stress generates microcracks within the hydrogel matrix. These cracks disrupt the sensor’s conductive pathways, leading to intermittent signal interruptions or complete loss of motion data, thereby preventing accurate recording of the entire movement process [14,15]. Environmental instability affects long-term reliability [16]. For instance, in high-humidity environments, conventional hydrogels absorb excessive moisture and swell, disrupting their internal network structure and reducing conductivity. These problems constitute fundamental bottlenecks at the material level and further restrict wearable devices’ comprehensive performance [17,18,19].

The emergence of conductive composite hydrogels provides an ideal solution to these problems [20,21]. As a type of soft material with a three-dimensional network structure and high water content, it inherently has advantages such as tissue-like softness, excellent stretchability, and biocompatibility. By introducing conductive fillers into the hydrogel matrix, a functional system integrating tunable conductivity, mechanical robustness, and biocompatibility can be constructed [22,23]. They can form a low-impedance interface with human skin and stably collect various physiological and motion signals [24]. Specifically, on the basis of retaining biocompatibility, they ensure efficient signal conduction and high-performance sensing properties through adjustable conductivity and mechanical properties, thus improving the signal quality [25,26]. On the other hand, they can adapt to human movement deformation through high stretchability and reliability, alleviating the situation of mechanical fragility [27,28]. The service life and stability could also be greatly improved through self-healing ability. These performance advantages make conductive composite hydrogels an ideal material choice for E-skins [23,29,30]. The development of composite conductive hydrogel is highly desirable for promoting more reliable and efficient applications of wearable E-skins, whether in high-precision physiological monitoring or HMI applications [31,32].

**Figure 1:**
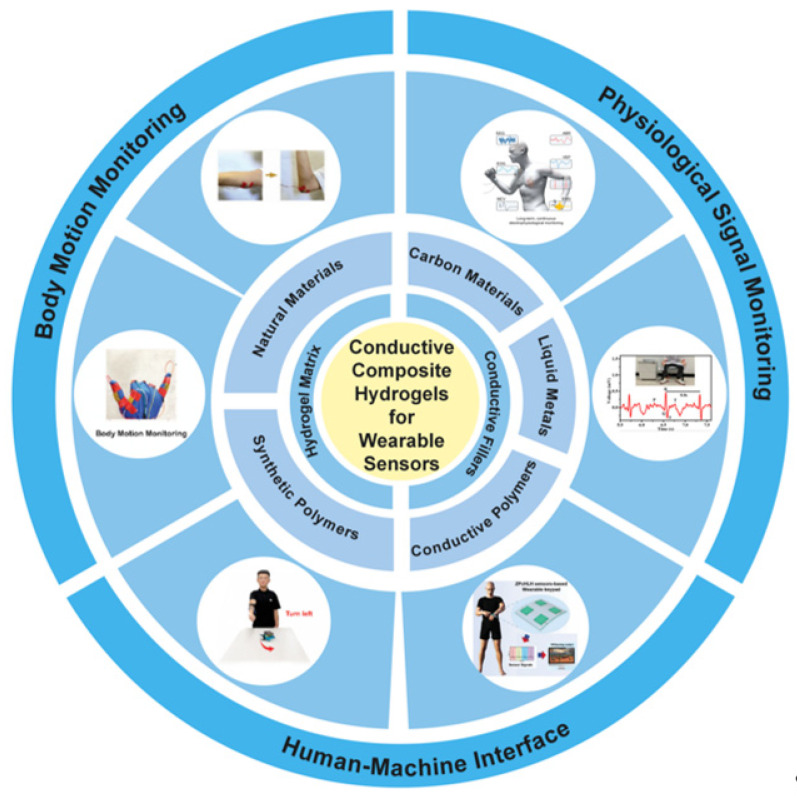
Diagram illustrating the material system and application scenarios of conductive composite hydrogels in the field of E-skins. Image for ‘Physiological Signal Monitoring’: Reproduced with permission. Ref. [33], Copyright 2024, AAAS. Image for ‘Physiological Signal Monitoring’: Reproduced with permission. Ref. [34], Copyright 2021, American Chemical Society. Image for ‘HMI’: Reproduced with permission. Ref. [35], Copyright 2023, Wiley-VCH. Image for ‘HMI’: Reproduced with permission. Ref. [36], Copyright 2024, Wiley-VCH. Image for ‘Body Motion Monitoring’: Reproduced with permission. Ref. [37], Copyright 2024, Wiley-VCH.

In this review, the latest advancements in conductive composite hydrogels for E-skin applications are discussed from three perspectives. As shown in Figure 1, the first section discusses the main hydrogel materials for preparing conductive composite hydrogel wearable sensors, including synthetic polymers and natural materials. The second section summarizes the typical conductive filler materials for constructing the unique functional systems of conductive composite hydrogels. Subsequently, the application scenarios of E-skins based on conductive composite hydrogels are reviewed, including electrophysiological signal monitoring, HMI, and body movement monitoring. Finally, based on the current trends in the development of E-skins using conductive composite hydrogels, a summary and outlook for their future development is discussed, hoping to offer guidance for advancing the development of conductive composite hydrogel-based E-skin sensors.

## 2. Materials for Preparing Hydrogels

With the rapid development of flexible electronics technology, wearable electronic skins have become the core carriers in the fields of health monitoring, motion tracking and HMI due to their lightweight, high elasticity and biocompatibility [38,39]. Among them, hydrogel has become an ideal choice for flexible substrates in wearable electronic devices due to its unique three-dimensional network structure, high water absorption and environmental responsiveness [40,41]. According to the source of raw materials, hydrogels can be classified into synthetic polymer hydrogels and natural material hydrogels [42], both of which have their own advantages in terms of performance, functionalization and application scenarios. The former is obtained from synthetic materials [31] through polymerization reaction, such as polyacrylamide(PAM), polyethylene glycol(PEG), etc., which is characterized by good structural controllability, good reproducibility, and excellent mechanical properties. The latter is prepared from natural materials [43], such as proteins, polysaccharides, natural glycyrrhetinic acid(GA), phytanic acid(PA), etc., which is characterized by good biocompatibility and biodegradability [44].

### 2.1. Synthetic Polymers

In response to the stringent requirements for high sensitivity, environmental adaptability, and long-term stability imposed by E-skins on materials [45,46], hydrogels have emerged as an ideal substrate for constructing such sensors due to their water content similar to human tissue, excellent biocompatibility, and tunable mechanical flexibility. However, the inherent limitations of pure aqueous hydrogels in terms of mechanical strength, conductivity, and environmental stability make it challenging to directly meet the demands of practical applications. In this context, conductive composite hydrogels constructed by introducing synthetic polymers have gradually become a research hotspot. These materials, through molecular design of the polymer network and synergistic regulation of functional components, which often rely on key molecular interactions such as hydrogen bonding, coordination complexes, and dynamic covalent bonds, not only inherit the biocompatible properties of hydrogels but also significantly enhance their mechanical performance, conductive stability, and environmental adaptability. These dual-action interactions form the cornerstone of performance enhancement: they act as reversible crosslinking points to dissipate energy and enhance mechanical toughness, while simultaneously improving interfacial compatibility and establishing hybrid conduction pathways enabling both ionic and electronic transport within the composite matrix to facilitate efficient charge transport. This provides a key solution for the high-performance and practical application of E-skins [31,47]. Commonly used synthetic polymers for the preparation of conductive composite hydrogels include polyvinyl alcohol (PVA) [48], polyacrylamide (PAAm), polyacrylic acid (PAA) [34] and so on.

**Figure 2:**
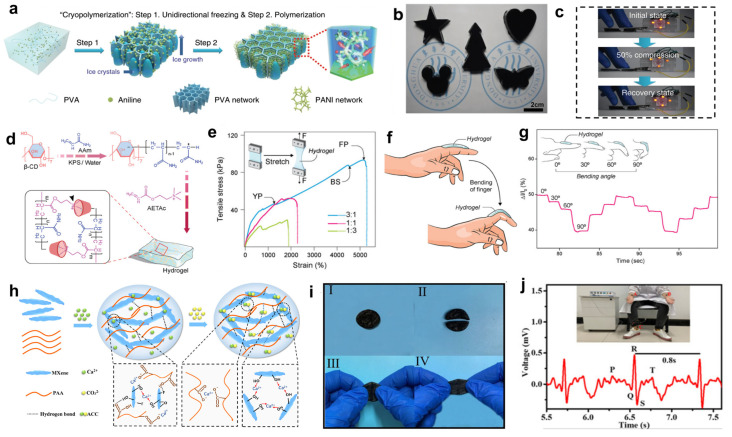
Synthetic polymers in conductive hydrogels for wearable sensor applications. (**a**–**c**) Low-temperature polymerization was used to prepare multi-shaped anisotropic PVA/polyaniline hydrogels (APPH), which were integrated into all-solid-state supercapacitors (A-SC) capable of stably illuminating LED arrays under compression/recovery deformation. Reproduced with permission from Ref. [48], Copyright 2020, Springer Nature. (**d**–**g**) β-CD-g-(pAAm/pAETAc) hydrogel was synthesized by radical polymerization, and its tunable mechanical properties enabled the fabrication of a sensor that adheres to the finger and accurately outputs step-like electrical signals corresponding to joint movements. Reproduced with permission from Ref. [54], Copyright 2024 Wiley-VCH GmbH. (**h**–**j**) By mixing MXene nanosheets with a PAA-ACC network, a hydrogel was prepared that can self-heal and restore its extensibility after breaking and can be used as a skin electrode to collect clear ECG signals. Reproduced with permission from Ref. [34], Copyright 2021, American Chemical Society.

As an important synthetic polymer with a large number of hydroxyl groups (-OH) on the molecular chain, PVA exhibits the core features of high hydrophilicity, excellent film-forming properties, adjustable mechanical properties, good biocompatibility, and flexibility of chemical modification in the field of conductive composite hydrogel-based E-skins [49,50,51]. Li et al. [48] proposed a low-temperature polymerization strategy for the preparation of anisotropic polyvinyl alcohol/polyaniline hydrogels (APPHs), which exhibited a bicontinuous phase structure consisting of ion-conducting PVA and electrochemically active polyaniline (PANI) scaffolds with high mechanical strength and superelasticity. Figure 2a illustrates the preparation process of APPH. A mixture of PVA, aniline, and initiator in an aqueous solution is frozen in a unidirectional vertical gradient, causing ice crystals to grow in a directed manner and form a 3D honeycomb structure. Under these low-temperature conditions, aniline undergoes restricted polymerization at the interface between ice crystals and PVA walls. Due to the low temperature inhibiting the reaction rate, PANI nanofibers slowly form a scaffold, ultimately yielding APPH material composed of an interpenetrating network of PVA and PANI. As shown in Figure 2b, with the advantages of easy operation and controllability of the low-temperature polymerization process, APPH materials with different shapes such as stars, hearts, trees, Mickey, butterflies, and so on, can be easily prepared. This can more conveniently achieve customized processing and patterned preparation. These anisotropic polyvinyl alcohol/polyaniline hydrogels can serve as multifunctional electrodes for the fabrication of all-solid-state supercapacitors (A-SCs). As the integrated device shown in Figure 2c consists of four A-SCs connected in series, the supercapacitor is able to simultaneously light up four light-emitting diodes (LEDs) even when subjected to 50% strain deformation, demonstrating good stability and practicality. The all-solid-state supercapacitors fabricated from anisotropic polyvinyl alcohol/polyaniline hydrogels can synchronously withstand stretching, bending, and other deformations alongside sensors while providing sustained and stable electrical energy, ensuring that sensors operate efficiently and continuously in applications such as motion monitoring and health tracking. This advancement drives the development of wearable electronic systems toward higher integration and reliability.

PAAm, as a common synthetic polymer, shows many unique advantages in the field of hydrogel preparation [52]. PAAm contains a large number of amide groups on the molecular chain, which have strong hydrophilicity, enabling PAAm to be fully dissolved in water to form a stable hydrogel system. In terms of mechanical properties, covalent crosslinked networks can be formed as the elastic skeleton, giving the hydrogel good tensile and elastic properties [53]. Arpita Roy and her team [54] designed a pseudo-slip ring β-CD-g-(pAAm/pAETAc) hydrogel based on adhesive and conductive supramolecular polymeric network (SPN), which combines the desired mechanical strength, excellent adhesion properties, Self-healing ability, and suitable electrical properties. The synthesis steps are shown in Figure 2d. The hydrogel was successfully produced by graft copolymerization via a one-pot method using potassium persulfate to initiate free radical polymerization with flexible Acrylamide (AAm), conductive poly 2-(acryloyloxy)ethyltrimethylammonium chloride(AETAc) and macrocyclic β-cyclodextrin(β-CD) as raw materials. By adjusting the AAm/AETAc ratio, all the physicochemical properties of the hydrogels could be adjusted. As shown in Figure 2e, the tensile strain curves of hydrogels at different AAm/AETAc ratios were demonstrated, and the addition of PAAm effectively enhanced the strain characteristics of hydrogels. To fabricate a pressure-sensing device based on β-CD-g-(pAAm/pAETAc) hydrogel, two electrodes were connected to each end of the hydrogel patch and connected to an electrochemical workstation. The engineered hydrogel-based sensor can be attached to different parts of the body to detect various human movements, specifically by monitoring changes in electrical signals and current. Figure 2f shows the sensor attached to the index finger, where it can detect angle changes from 0° to 30°, 60°, and 90°, generating the staircase-like current curve shown in Figure 2g, demonstrating the sensor’s rapid response performance and excellent repeatability. This engineered sensor demonstrates higher efficiency, stability, and sensitivity in motion/tactile sensing, advancing real-time human healthcare monitoring.

PAA [55,56] is a water-soluble polymer with good biocompatibility and contains a large number of hydrophilic -COOH groups, which can confer high electrical conductivity to the hydrogel. The high content of carboxyl groups enhances the stability of the active substance-electrode connection and improves the mechanical properties of the sensor [57,58,59]. Li et al. [34] prepared a self-healing, degradable, and conductive composite hydrogel for multifunctional epidermal sensors. Figure 2h illustrates the principle behind the preparation of the hydrogel. The MXene-PAA-ACC hydrogel is inspired by the biomineralization process and is prepared by combining inorganic components with an organic matrix. Specifically, simply incorporating MXene nanosheet network into the mixed network of PAA and amorphous calcium carbonate (ACC) yields a hydrogel with excellent degradability, elasticity, and self-healing capabilities. This hydrogel can be applied to fields such as wearable sensors and tissue engineering. In Figure 2i, step I is the original hydrogel, step II is the completely fractured hydrogel, step III is the result of the fractured hydrogel after self-healing at room temperature, and step IV shows that the Self-healed hydrogel undergoes stretching and does not break, which demonstrates that the hydrogel has a strong self-healing ability. As shown in Figure 2j, the MXene-PAA-ACC hydrogel can be used to fabricate epidermal electrodes for detecting electrocardiogram (ECG) signals. By attaching the electrode to the forearm of the tester and connecting the grounded electrode to the ankle, clear P, Q, R, S, and T waves were successfully measured, and the heart rate of the tester was 75 beats per minute, which is in the normal range for adults, calculated from the interval of 0.8 s between two P waves, highlighting the potential of the MXene-PAA-ACC hydrogel for use in electrophysiological monitoring and personal health management. The sensor made from this hydrogel combines reliable sensing, ultra-fast response, excellent self-healing, and biodegradability, offering an innovative solution for electronic skin, personalized health monitoring, and HMI devices.

### 2.2. Natural Polymer Materials

In the selection of materials for E-skins, natural materials are becoming the focus of research with their unique advantages over synthetic polymers [60]. Unlike synthetic materials, these materials are mainly extracted and obtained from living organisms such as plant and animal tissues, microbial metabolites, etc., such as sodium alginate, chitosan, gelatin and PA. In view of the stringent requirements for comfort, safety and environmental adaptability of wearable devices, the biodegradability, green renewability, excellent dermal compatibility, and low preparation cost of natural materials have been attracting attention in recent years [43].

**Figure 3:**
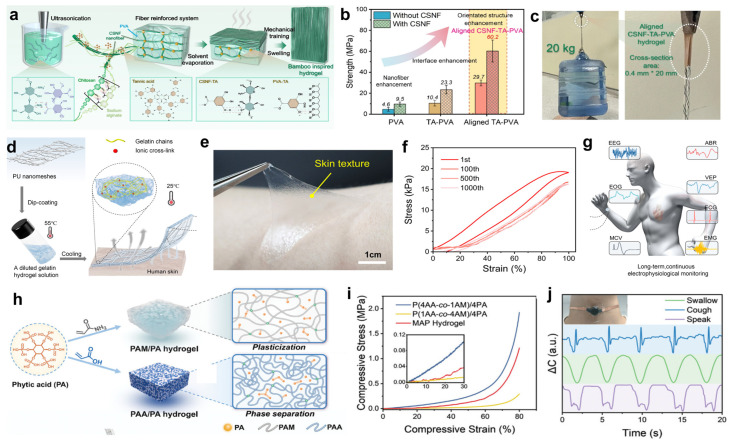
Natural materials in conductive composite hydrogels for wearable sensor applications. (**a**–**c**) CSNF-TA-PVA composite hydrogel was prepared through bamboo-inspired structural design, with a strength of 60.2 MPa, capable of withstanding a 20 kg load (equivalent to 50,000 times its own weight) without structural damage. Reproduced with permission from Ref. [62], Copyright 2025, Springer Nature. (**d**–**g**) Ultra-thin hydrogels were prepared using a PU nano-network-enhanced design, which combines strong adhesion/high elasticity with fatigue resistance (after 1000 cycles of 100% strain), enabling long-term stable monitoring of various electrophysiological signals. Reproduced with permission from Ref. [33], Copyright 2024, The American Association for the Advancement of Science. (**h**–**j**) By differentially regulating PAM/PA and PAA/PA hydrogel networks through phytic acid (PA), MAP hydrogels with high mechanical properties (stress reaching 1.2 MPa at 80% strain) were obtained, which can detect physiological activities such as swallowing and coughing in real time. Reproduced with permission from Ref. [63], Copyright 2025, Wiley-VCH GmbH.

Chitosan and sodium alginate [61], as natural polysaccharides, have excellent flexibility and stretchability, which can closely fit the human skin and follow the various deformations of the skin without rupture or detachment, ensuring that the sensors always maintain a stable monitoring performance during complex human movement. Inspired by bamboo, Zhuo et al. [62] developed a fiber-reinforced composite hydrogel, the preparation principle is shown in Figure 3a, using PVA as the hydrogel matrix and TA as the cross-linking agent, chitosan–sodium alginate nanofibers (CSNFs) were prepared by using strong electrostatic interactions between two polysaccharides with oppositely charged groups, firstly, mixing, casting and drying of the CSNFs with the PVA solution, and then subsequently, placing the resulting materials were placed in TA solution and rehydrated to induce TA penetration and cross-linking of the composites to complete the preparation of CSNF-TA-PVA hydrogels. The –OH groups of TA could potentially form molecular interactions with the –${NH}_{2}$ groups of CSNFs and the –OH groups of PVA. As shown in Figure 3b, the strength of the aligned CSNF-TA-PVA hydrogel soared to 60.2 MPa by over-hardening after stretching and immobilizing it in a high-concentration salt solution. Figure 3c shows that even a small piece of composite hydrogel can easily lift a 20 kg object—equivalent to 50,000 times its own weight—while maintaining its structural integrity throughout the process without any signs of fracture, demonstrating the hydrogel’s extremely strong tensile properties. Whether used as a flexible sensor for monitoring joint movement or as a motion capture device attached to muscle surfaces, this hydrogel maintains structural stability and reliable sensing performance under high-intensity use, avoiding monitoring interruptions caused by material damage. This outstanding load-bearing capacity and structural robustness directly translate into E-skin’s excellent durability, ensuring that the sensor maintains mechanical integrity and reliable signal output, even when subjected to extreme stretching or unexpected impacts during complex human movements, thereby preventing easy failure. It has the potential to overcome the limitations of existing materials, adapt to high-intensity training and dance capture scenarios, extend the lifespan of sensors, and promote the development of electronic skin towards greater durability, efficiency and wider application.

Gelatin [64,65], as a hydrolyzed product of collagen, is derived from animal skin, bone, etc. It has excellent biocompatibility and can be decomposed by microorganisms in the natural environment. By compounding it with other polymers, gelatin can build a dual network structure, which can significantly enhance the mechanical strength of the hydrogel, and it also shows significant advantages in terms of environmental adaptability, functional integration, and preparation process. Zhang et al. [33] developed a polyurethane (PU) nano-network-enhanced breathable hydrogel sensor capable of continuous health monitoring for 8 days under everyday conditions. The preparation principle of the PU nano-network-enhanced hydrogel is shown in Figure 3d, where the ultra-thin PU nano-network is simply immersed in a thermally dependent phase-change hydrogel solution of gelatin. Despite its thickness of only approximately 10 μm, the hydrogel exhibits excellent stretchability, skin adhesion, and breathability. Figure 3e shows a photograph of the ultra-thin hydrogel being peeled off human skin, clearly demonstrating its superior strong adhesion and high elasticity: it remains tightly adhered to the skin even under stretching deformation and does not break or leave residue even when forcibly torn. As illustrated in Figure 3f, the ultra-thin hydrogel can withstand 1000 stretch-release cycles at 100% strain. A noticeable hysteresis loop appears during the first loading/unloading due to viscoelastic energy dissipation, but subsequent stress curves overlap almost perfectly, indicating excellent fatigue resistance. It can be seen from Figure 3g that this ultra-thin hydrogel can be used for long-term, continuous electrophysiological monitoring, covering various physiological signals such as ECG, electromyogram (EMG), motor nerve conduction velocity (MCV), electrooculogram (EOG), EEG, auditory brainstem response (ABR), and visual evoked potential (VEP). This means it can comprehensively capture human physiological state information, providing comprehensive data support for health management, disease early warning, and rehabilitation monitoring. Whether used for real-time physical performance monitoring of athletes, daily health management of chronic disease patients, or tracking the recovery status of post-surgical rehabilitation populations, this hydrogel sensor demonstrates significant potential for wearable applications, with the potential to drive the development of wearable medical devices toward lighter, more comfortable, more precise, and longer-lasting designs.

PA [66], a naturally occurring strongly acidic organophosphonic acid compound, exhibits significant advantages in the preparation of E-skins from natural hydrogels due to its unique chemical structure and biocompatibility. The main features include high electrical conductivity, frost resistance, moisturization, antimicrobial properties, high transparency, tunable mechanical properties, and environmental stability, which together enhance the sensitivity, reliability, and range of applicability of the sensors [63,67]. Song et al. [63] developed a phytic acid-induced gradient hydrogel for high-sensitivity and wide-range pressure sensing. As illustrated in Figure 3h, in PAM/PA hydrogels, PA plasticizes PAM, reducing its mechanical strength, while in PAA/PA hydrogels, PA causes PAA to phase separate and enhances the hydrogel, indicating that PA molecules cause different hydrogel networks to undergo different interactions, resulting in distinct mechanical property changes. Equal volumes of AM/PA precursor solution and AA/PA precursor solution (400 μL each) were selected. The AA/PA solution was subjected to 20 min of Ultraviolet(UV) pre-exposure treatment, and the resulting hydrogel was denoted as MAP gradient ion hydrogel. Figure 3i demonstrates that the MAP hydrogel exhibits excellent mechanical properties. When the compressive strain reaches 80%, the compressive stress of the MAP hydrogel reaches 1.2 MPa. As shown in the inset of the figure, the compressive stress of the MAP hydrogel continues to increase once the compressive strain exceeds 10%, demonstrating excellent mechanical performance. As shown in Figure 3j, the combination of physical objects and waveforms clearly demonstrates that flexible sensors can identify signals associated with swallowing, coughing, and vocalization, with distinct waveform characteristics for each. These signal differences provide data support and visual evidence for the research and application of physiological monitoring technology. This functional hydrogel, which exhibits outstanding sensing performance and biological activity, shows extraordinary potential in wearable sensing applications.

In summary, synthetic polymer hydrogels and natural material hydrogels each have their own advantages in the field of wearable electronic devices. The choice between the two is not a matter of superiority or inferiority, but rather depends on the specific requirements of the target electronic skin application. It typically involves balancing trade-offs between mechanical strength, electrical properties, and biocompatibility. When high mechanical strength, excellent and tunable conductivity, and environmental stability are the primary considerations, synthetic polymer hydrogels are generally the preferred materials. Their structurally controllable networks and excellent reproducibility make them ideal for applications requiring robust and reliable performance under repetitive mechanical stress. This makes them particularly suitable for demanding applications such as high-precision joint motion tracking, human–machine interfaces requiring rapid response, or wearable energy devices. Conversely, when exceptional biocompatibility, biodegradability, and minimal environmental impact are preferred, natural material hydrogels are the ideal choice. Their innate affinity with biological tissues reduces the risk of inflammation and enables safe, long-term integration with the skin. Therefore, natural material hydrogels [68] are the preferred foundation for applications such as long-term, continuous health monitoring patches, implantable bioelectronics, and environmentally friendly disposable sensors. In these contexts, the superior safety profile and sustainability of natural materials often outweigh the need for extreme mechanical robustness or the highest possible conductivity. Through the optimization of different material properties and preparation processes, both categories of hydrogels jointly promote the development of E-skins, providing diversified and scenario-specific material solutions for health monitoring, human–machine interaction, and other fields [69].

## 3. Conductive Filler Materials

Wearable electronic devices are characterized by their flexibility, stretchability, and adaptability. In recent years, they have attracted considerable attention due to their potential in digitalization and changing personal lifestyles [70]. Hydrogel, as a class of soft materials with both high water content and network structure, can build a functional system with flexibility, conductivity and biocompatibility through the introduction of conductive filler components, becoming an ideal medium for connecting human physiological signals and electronic devices [71]. Currently, carbon materials (carbon nanotubes [72], graphene [73], carbon fibers [74]), liquid metals (such as eutectic gallium indium alloy (EGaIn)) and conductive polymer materials polypyrrole (PPy), polyaniline (PANI), poly(3,4-ethylenedioxythiophene): polystyrene sulfonate (PEDOT:PSS) due to the unique electrical properties and mechanical suitability, the core choice of hydrogel conductive filler, and its structural design and performance regulation directly determines the sensor’s sensitivity, stability, and application scenarios [75,76,77]. The selection criteria are application-driven: carbon materials offer superior conductivity (>10^3^ S/m) for high-sensitivity sensors, liquid metals provide self-healing capability with stable conductivity under deformation, while conductive polymers enable biocompatible interfaces with tunable conductivity (10^−3^–10^3^ S/m). Sensor performance depends on the conductive network mechanism, particularly the percolation threshold and pathway evolution during deformation. The common performances of hydrogels prepared with different types of conductive fillers are summarized in Table 1.

### 3.1. Carbon Materials

Carbon material has become the core choice of hydrogel conductive filling for E-skins due to its excellent electrical properties, mechanical properties and biocompatibility [85,86]. However, they still face two key limitations: first, their strong hydrophobicity tends to weaken interfacial bonding with hydrophilic hydrogels. Second, the high cost of producing high-purity graphene requiring precision manufacturing, to some extent restricts large-scale application. Despite these limitations, carbon materials retain prominent core advantages. Their intrinsic high conductivity and stable environmental response capability enable the precise capture of tiny human physiological signals and large-deformation movements. Moreover, they combine high strength with flexibility, making them suitable for complex scenarios such as joint bending and electronic skin. Additionally, they possess advantages like low cytotoxicity and skin compatibility. These strengths allow carbon materials to demonstrate potential for large-scale applications in the field of intelligent health monitoring, holding promise to drive wearable devices toward greater precision, comfort, and multifunctionality [87,88].

**Figure 4:**
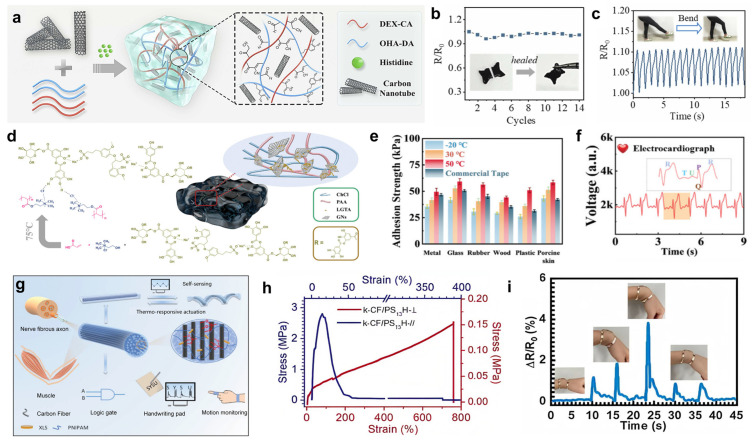
Advanced preparation strategies for conductive carbon-filled hydrogels. (**a**–**c**) By directing the internal structure of CNT hydrogels, they can be made to be both conductive and self-healing, enabling real-time monitoring of changes in resistance during knee flexion. Reproduced with permission from Ref. [72], Copyright 2023, Elsevier Ltd., Amsterdam, Netherlands. (**d**–**f**) Based on its unique microstructure design, LTGH exhibits high adhesion strength in a wide temperature range of −20 to 50 °C and can be used as a physiological electrode for accurate heart rate detection. Reproduced with permission from Ref. [73], Copyright 2025, Wiley-VCH GmbH. (**g**–**i**) Anisotropic carbon fiber composite hydrogels were prepared through biomimetic design, and their differentiated mechanical properties in parallel/perpendicular fiber directions can be integrated into flexible sensors to monitor wrist bending signals in real time. Reproduced with permission from Ref. [74], Copyright 2022, Wiley-VCH GmbH.

Carbon nanotubes (CNTs) are one-dimensional nanomaterials formed by curling single-layer or multi-layer graphene sheets, exhibiting excellent electrical properties, good mechanical strength, and high specific surface area. As a filler composite with hydrogel, it can not only significantly improve the electrical conductivity of the hydrogel, but also enhance its mechanical properties, and endow the composite with a unique sensing performance [33,89,90,91]. In recent years, CNT-filled hydrogel-based E-skins have shown great potential in human motion monitoring, physiological signal detection, etc., and related research results continue to emerge. Ding et al. [72] designed and prepared a new type of self-healing CNT-based hydrogel for the detection of human motion, which can be triggered by the stimulus to realize the on-demand movement function of the wearable flexible electronic device. The preparation principle and structure are shown in Figure 4a: dopamine-modified oxidized hyaluronic acid (OHA-DA), dextran functionalized with cyanoacetic acid (DEX-CA), and CNTs were mixed and crosslinked into a gel catalyzed by histidine. The resulting hydrogel not only possesses strong tissue adhesion but also converts motion stimuli into electrical resistance signals. In addition, the photothermal conversion property of CNTs gives the hydrogels NIR-driven mobility. Figure 4b demonstrates the strong self-healing ability of CNT hydrogels, where the resistance value of the hydrogel is essentially stable even after several thorough fracture/healing cycles. It can be seen from Figure 4c that CNT hydrogels can withstand significant deformation, enabling them to detect human leg movements. The CNT-based system outperforms alternative fillers with superior deformation tolerance, while the NIR-triggered mobility enables remote sensor repositioning and on-demand dissolution, addressing practical challenges in long-term E-skin applications. The hydrogel’s resistance changes in response to leg movement patterns. Converting various human movements into resistance signals demonstrates the broad applicability of this CNT-based hydrogel in bioelectronic interfaces. Additionally, the hydrogel can be dissolved upon exposure to near-infrared (NIR) light, enabling on-demand mobility. This trigger-activated removable property offers new design concepts for next-generation wearable devices and provides innovative strategies for addressing the challenge of removing bioelectronic devices, highlighting its unique advantages and potential in the field of E-skins.

Graphene, as a carbon material with a single atomic layer two-dimensional honeycomb lattice structure, has become an ideal filler for hydrogel composite modification by virtue of its ultra-high carrier mobility, excellent mechanical strength, large specific surface area, and rich surface chemistry. The introduction of graphene into the hydrogel system can not only build an efficient conductive network and significantly improve the electrical properties of composites, but also enhance the mechanical toughness and environmental stability of hydrogels [90,92]. In recent years, graphene-filled hydrogel-based E-skins have become the focus of research in the field of flexible sensing as they have demonstrated excellent performance in application scenarios such as pulse monitoring, muscle electrical signal capture, and pressure sensing [93,94,95]. Wang and his team [73] developed a multifunctional lignin–tannin nanorod graphene-doped hydrogel (LTGH) for wearable flexible pressure sensors. Figure 4d reveals that the LTGH preparation process: deep eutectic solvent (DES) was formed in ethylene glycol, potassium persulfate (initiator) and methylenebisacrylamide (cross-linking agent) were added, and the graphene was dispersed through self-assembled nanospheres of sodium lignosulfonate and tannic acid, and LTGH was produced by polymerization. The LTGH obtained combines excellent conductivity, wide sensing range, high sensitivity, strong adhesion, antimicrobial and UV resistance, and can work stably at −80 to 50 °C after doping with ethylene glycol. Figure 4e shows the adhesion of LTGH on the surface of different substances at −20 °C, 30 °C and 50 °C, which have reached or exceeded the market standard. As illustrated in Figure 4f, LTGH can accurately identify P, Q, R, and T waves, laying a solid foundation for continuous and reliable monitoring of core human health indicators. Its high performance, all-weather operational capability, excellent signal quality, and long-term stability make it an ideal choice for developing next-generation smart patches, smart clothing, and even implantable devices. This will enable truly seamless, continuous, and precise cardiovascular health monitoring, extending from hospitals to homes and daily life, providing powerful tools for preventive medicine and personalized health management, marking its emergence as a revolutionary material in the field of physiological sensing.

Carbon fiber (CF) is a micron-sized filamentary material formed from organic fibers (e.g., polyacrylonitrile) by high-temperature carbonization and graphitization. As a high-performance carbon-based material, it combines ultra-high strength, light weight and electrical conductivity, which has become the key to break through the bottleneck. Filling it into the hydrogel can enhance the material’s deformation resistance as well as build a conductive network, which significantly improves the sensitivity and durability of the sensor [74,88]. Inspired by anisotropic biological tissues, Li et al. [74] developed anisotropic carbon fiber composite hydrogels with excellent mechanical properties and conductivity for logic gates, integrated soft actuators, and sensors with ultra-high sensitivity. As shown in Figure 4g, inspired by the directional signal transmission of nerve fiber axons and the directional contraction of muscle fibers, a material was constructed using carbon fibers and cross-linked poly-N-isopropylacrylamide (XLSPNIPAM). The carbon fibers provide high electrical conductivity and strain sensitivity, while XLSPNIPAM exhibits thermoresponsive reversible deformation capabilities. This forms an anisotropic structure with high performance in the parallel direction and lower performance in the perpendicular direction, enabling self-sensing and thermoresponsive drive functions. The material can be applied to scenarios such as logic gate construction, touchscreens, and motion monitoring, providing high-performance solutions for related fields. Figure 4h shows the stress–strain curves of the CF composite hydrogel in the parallel and perpendicular directions. When the hydrogel is stretched in the parallel direction, the strain of the CF composite hydrogel is approximately 15% and the yield strength is about 3 MPa. After that, as the strain rate increases, the stress decreases sharply until the hydrogel breaks. When the hydrogel is stretched in the perpendicular direction, within a strain of 700%, the stress of the CF composite hydrogel increases with the increase in strain at a nearly linear rate. After reaching a certain critical point, the stress reaches a maximum value of 0.15 MPa, and then the hydrogel breaks, with the stress dropping almost vertically to 0. It is shown that the highly aligned k-CF causes the composite hydrogel to exhibit strong anisotropy in mechanical properties. As depicted in Figure 4i, CF composite hydrogels can be used to make E-skins that detect human movement. When the wrist is bent at angles of 30°, 45°, and 55°, different electrical signals are generated, and when the wrist returns to 0°, the electrical signals immediately disappear. This hydrogel shows broad application prospects in cutting-edge fields such as flexible devices.

### 3.2. Liquid Metals

Room temperature liquid metals (LMs) [96], especially EGaIn, are highly promising and revolutionary filler media due to their unique metal-grade electrical conductivity, inherent mobility, low toxicity, and excellent deformation capabilities. Embedding EGaIn micro/nanodroplets or structured patterns into a hydrogel matrix not only builds efficient electron transport pathways and significantly improves electrical conductivity and sensing sensitivity, but also confers synergistic enhancement of composites, such as dynamic self-healing, extreme tensile tolerance, and multimodal stimulation response. However, challenges remain, including the spontaneous oxidation of the EGaIn surface that may affect long-term conductivity stability, and potential biocompatibility concerns from Ga/In ion leakage during prolonged skin contact, necessitating appropriate encapsulation strategies. Despite these limitations, this liquid metal–hydrogel intelligent composite system opens up a new way for the development of a new generation of high-performance, highly comfortable, long-term wearable flexible electronic sensors, showing great potential in personalized health monitoring and human–machine interface and other cutting-edge fields [97,98,99].

**Figure 5:**
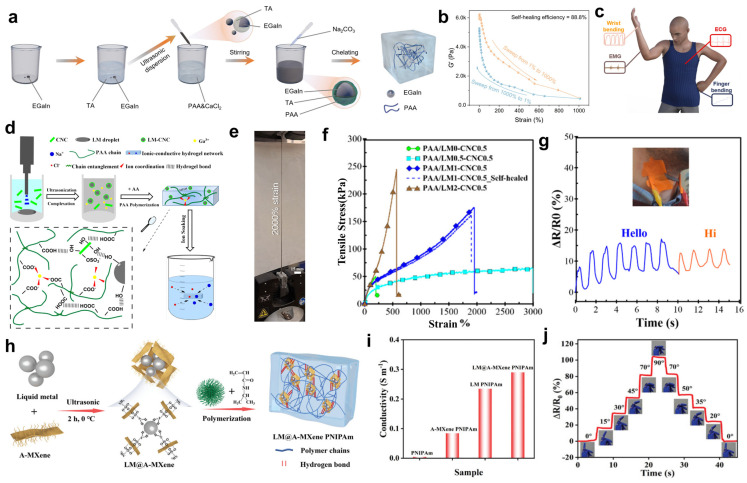
Advanced engineering of EGaIn liquid metal for multifunctional soft electronics. (**a**–**c**) PAA-ACC-LM@TA multilevel structure hydrogel formed through room temperature self-assembly, whose rheological recovery experiment proved that the G′ recovery rate reached 100% after 1000% strain, and can be integrated into human health monitoring sensors. Reproduced with permission from Ref. [79], Copyright 2025, American Chemical Society. (**d**–**g**) By synthesizing LM-CNC composite hydrogels, which have extreme stretchability of 2000% (initial 15 mm stretched to 300 mm) and mechanical properties that can be adjusted according to LM content, human movement signals such as speech can be monitored in real time. Reproduced with permission from Ref. [80], Copyright 2024, American Chemical Society. (**h**–**j**) By preparing stretchable conductive hydrogels, in which the LM@A-MXene composite has the best conductivity, it can respond in real time to changes in resistance caused by finger bending. Reproduced with permission from Ref. [81], Copyright 2023, Wiley-VCH GmbH.

EGaIn, serving as a filling medium in hydrogels, offers a groundbreaking solution to overcome the durability limitations of traditional conductive hydrogels [100]. Its core advantages stem from its inherent fluidity and dynamic self-healing properties: when microcracks form in the hydrogel network due to external forces, EGaIn droplets can autonomously flow and fill defects, rapidly restoring conductive pathways. Additionally, its extremely low shear modulus efficiently disperses stress, with both mechanisms collectively enhancing the hydrogel’s cyclic durability [101,102]. In response to the problem of unstable signal acquisition of commercially available Ag/AgCl gels in rehabilitation exercises, Wei et al. [79] developed a liquid metal-based multistage hydrogel, which guarantees the stable acquisition of electrophysiological signals during exercise with the characteristics of adhesion, self-healing, high stretch and conductivity, etc. As shown in Figure 5a, the synthesis principle of the hydrogel is as follows: EGaIn and TA are first added to deionized water, followed by ultrasonic treatment to form a uniformly dispersed EGaIn@TA dispersion solution that remains stable without precipitation after 7 days of static incubation. The EGaIn@TA dispersion is then added dropwise to a mixture of PAA and calcium chloride (CaCl_2_) to form a hydrogel precursor. An equal volume of sodium carbonate (Na_2_CO_3_) solution is then added to crosslink the mixture, resulting in the final PAA-ACC-LM@T hydrogel (PATL). In Figure 5b, the self-healing efficiency of PATL1.5 hydrogel was evaluated by quantitative destructive testing: the strain was increased from 1% to 1000% and then decreased to 1%, at which time the stress was 5540 Pa, and the self-healing efficiency was calculated to be 88.8%. Figure 5c demonstrates the synchronous monitoring capability of the multifunctional wearable sensor assembled from LM-based hydrogels for multidimensional physiological and motion signals of the human body, including wrist joint bending movements, skin conductance activity (EGG/EDA), EMG, and finger joint bending. This visually demonstrates the integrated design of flexible sensors for limb motion tracking and bioelectric signal acquisition, highlighting their multi-parameter sensing advantages in HMI and health monitoring. It provides new insights into the application of smart hydrogels in health monitoring, rehabilitation training, and HMI fields.

The emergence of EGaIn, a liquid metal with unique physicochemical properties, such as excellent fluidity, high electrical conductivity, and good thermal stability, has brought a ray of hope for solving the problem of hydrogel toughness [97,98]. EGaIn has unique physicochemical properties, such as excellent fluidity, high electrical conductivity, and good thermal stability, etc. Its integration into E-skins as a hydrogel filler can significantly improve the toughness of hydrogels. On one hand, EGaIn interacts with the hydrogel polymer network through hydrogen bonds and coordination bonds, strengthening connections to uniformly distribute stress and prevent rupture. On the other hand, its high extensibility grants the hydrogel additional deformation capacity, enabling it to maintain structural stability by flowing and deforming under stress [103,104]. Pooria Rahmani and his team [80] came up with a PAA hydrogel prepared using cellulose nanocrystals (CNCs) stabilized LM particles as an initiator. This hydrogel not only possesses excellent toughness, but also has piezoelectric properties to generate high currents. The gel formation mechanism is shown in Figure 5d: Ultrasonic treatment breaks down the liquid metal into particles and releases Ga^3+^, while CNCs spontaneously coat the particle surfaces. Upon addition of monomers, the liquid metal initiates radical polymerization, and the carboxyl groups of the polymer backbone cross-link with CNCs or Ga^3+^ via hydrogen bonds/ionic bonds, forming a hydrogel. Figure 5e demonstrates the excellent tensile properties of the hydrogel, with a tensile strain reaching 2000%. Figure 5f compares stress–strain curves to reveal the effect of EGaIn liquid metal content on the mechanical properties of PAA/CNC hydrogel: the 2-part LM group (PAA/LM2-CNC0.5) exhibits the highest strength (nearly 250 kPa), and its performance is fully restored after self-healing, demonstrating that EGaIn filling can simultaneously optimize the hydrogel’s toughness, durability, and self-healing properties. The hydrogel sensor was installed on different parts of the volunteer’s body using only the inherent adhesion of the gel, with external electrodes secured with tape. Figure 5g shows the real-time dynamic response of the flexible sensor to two external stimuli (“Hello” and “Hi” spoken by the tester) via the resistance change rate–time curve. The sensor exhibits a significant resistance change (peak 40–50%) within 0.5 s after stimulus activation and fully recovers to baseline within 1 s, demonstrating high sensitivity, millisecond-level response speed, and excellent signal stability. This ensures the sensor’s continuous and reliable operation in complex mechanical environments, expanding its application in long-term, high-intensity usage scenarios. It is a strong candidate for wearable strain sensors, soft electronics, and self-powered touch sensors.

The introduction of the liquid metal EGaIn provides an innovative path to break through the bottleneck of hydrogel electrical conductivity, which is much higher than that of conventional hydrogel systems due to its room-temperature liquid behavior and excellent electron conduction ability [97,105,106]. On one hand, when EGaIn is incorporated as a filler into hydrogels, it can establish efficient electronic transport pathways within the polymer network, enabling an ion-electron hybrid conduction mechanism. On the other hand, the synergistic interaction between EGaIn and the hydrogel matrix ensures that the composite material maintains stable conductive pathways during deformation processes such as stretching and bending. This property not only enhances the sensitivity and response speed of E-skins but also ensures signal stability in dynamic monitoring scenarios, providing critical material support for the intelligent upgrading of wearable devices. [98,107] Ma and his team [81] developed and invented a highly stretchable and conductive hydrogel for the preparation of biomimetic self-sensing soft actuators. Figure 5h illustrates the fabrication process of the hydrogel: liquid metal and A-MXene are first taken and subjected to ultrasonic treatment at 0 °C to break the large liquid metal droplets into particles and interact with A-MXene, forming LM@A-MXene. Subsequently, monomers are added, and through polymerization reactions, utilizing interchain hydrogen bonds between polymer chains, a three-dimensional network structure of LM (A-MXene) Poly(N-isopropylacrylamide)(PNIPAm) hydrogel is constructed. As shown in Figure 5i, the interbridged LM@A-MXene nanodroplets form a 3D interconnected conductive network, enhancing the conductivity of the hydrogel. The conductivity of LM@A-MXene PNIPAm is the highest, reaching 0.3 S/m, followed by LM PNIPAm and A-MXene PNIPAm, with PNIPAm having the lowest conductivity. It can be seen from Figure 5j that the hydrogel can detect the human motion state. Under different finger bending angles (0°, 15°, 30°, 45°, 70°, 90°, etc.), the hydrogel exhibits different resistance values, fully demonstrating its excellent angular resolution, outstanding stretchability, and responsiveness. This makes it an efficient conductive material option for flexible sensors, wearable electronics, and other fields, potentially enhancing the detection accuracy and reliability of devices through its stable conductive performance, thereby accelerating the practical application of flexible electronic devices.

### 3.3. Conductive Polymers

Beyond inorganic conductive fillers such as carbon materials and liquid metals, conductive polymers (CPs) represent another distinct class of organic functional materials, offering a crucial pathway for constructing conductive composite hydrogels for E-skins. Among these, Polypyrrole (PPy), Poly(3,4-ethylenedioxythiophene)–polystyrene sulfonate (PEDOT:PSS), and Polyaniline (PANI) are among the most extensively studied representatives [108,109]. These polymers, owing to their conjugated π-electron backbone, enable efficient charge transport along the polymer chains, thereby exhibiting intrinsic electrical conductivity. Compared to inorganic fillers, CPs uniquely combine the conductive properties of traditional conductors with the advantages of polymeric materials, such as flexibility, low cost, ease of processing, and potential biocompatibility [110,111]. Furthermore, their tunable conductivity, reversible electrochemical doping/dedoping processes, relatively simple preparation methods, and environmental friendliness make them ideal candidates for developing high-performance conductive polymer hydrogels (CPHs), demonstrating immense potential, particularly in biosensing, neural interfaces, and electronic skin applications [112,113]. In recent years, researchers have continuously optimized the performance of CPHs through various strategies, ranging from molecular design to microstructure regulation, thereby promoting their development in wearable sensor applications [14,114,115].

**Figure 6:**
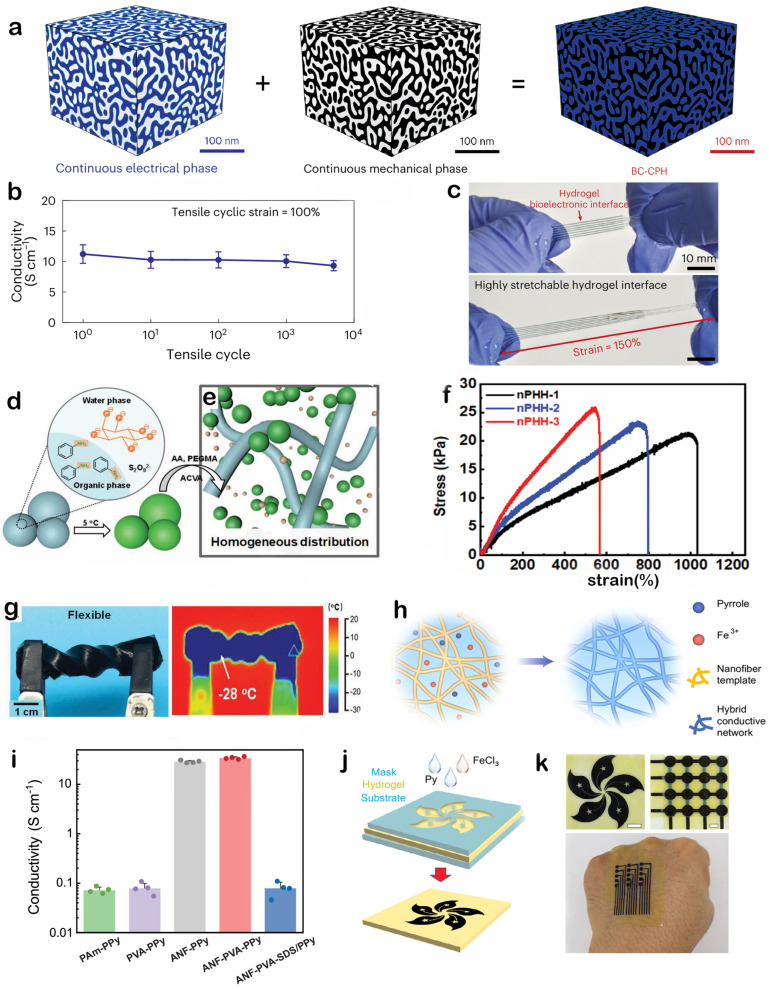
Advanced strategies for fabricating high-performance conductive polymer hydrogels. (**a**–**c**), A bi-continuous PEDOT:PSS/polyurethane hydrogel achieves a combination of high conductivity, stretchability, and cyclic stability. Reproduced with permission from Ref. [82], Copyright 2023, Springer Nature. (**d**–**g**) Uniformly dispersed nano-sized PANI particles are used to create an ultra-stretchable and anti-freezing conductive hydrogel**.** Reproduced with permission from Ref. [83], Copyright 2022, Wiley-VCH. (**h**–**k**) An aramid nanofiber template guides the in situ polymerization of PPy to form highly conductive and precisely patternable networks. Scale bars: 1 cm. Reproduced with permission from Ref. [84], Copyright 2023, Springer Nature.

To enhance the performance of conductive hydrogels, significant efforts have been directed towards creating materials that synergistically combine robust mechanical properties with high electrical conductivity. In this context, PEDOT:PSS has emerged as a key material. A notable advancement was reported by Zhou et al. [82], who developed a bi-continuous conductive polymer hydrogel (BC-CPH), as illustrated in Figure 6a. This material simultaneously achieved high electrical conductivity (over 11 S/cm), high stretchability (over 400%), and remarkable fracture toughness (over 3300 J/m) within physiological environments. The BC-CPH was prepared from phase-separated inks containing an electrical phase of PEDOT:PSS and a mechanical phase of hydrophilic polyurethane, dissolved in a mixed solvent of ethanol and water. An optimal PEDOT:PSS concentration (20–30 *w*/*w*%) was identified to ensure the bi-continuous presence of both phases, enabling the material to maintain high conductivity without sacrificing its mechanical integrity. This unique structure resulted in favorable electrical and electrochemical performance. For instance, the hydrogel sustained high electrical conductivity (over 11 S cm^−1^) even after undergoing 5000 cycles of 100% tensile strain, as shown in the data presented in Figure 6b. Furthermore, the all-hydrogel bioelectronic interface demonstrated exceptional durability, withstanding over 150% strain without fracture, a capability visualized in Figure 6c. The combination of these properties, along with its compatibility with advanced manufacturing methods like 3D printing, makes this hydrogel a highly promising candidate for fabricating complex, high-performance bioelectronic devices for applications in neural interfacing and wearable health monitoring.

To address the inherent brittleness and processing challenges of traditional PANI, an alternative strategy focusing on nano-engineering the conductive filler was developed. Yu et al. [83] reported a high-performance PANI-based conductive hydrogel, achieved by synthesizing nano-sized polyaniline particles and introducing them as conductive fillers into a polymer network. A key innovation of this work was the controllable synthesis of these PANI nano-particles through an ingenious multiphase reaction pathway, the process of which is schematically shown in Figure 6d. This laid the foundation for the subsequent construction of a high-performance composite material. Figure 6e further demonstrates a significant advantage of this approach: the nano-particulate form of PANI can be uniformly dispersed in the polymer matrix, effectively mitigating the aggregation issues often encountered with traditional PANI. This uniform dispersion is crucial for constructing continuous and effective conductive networks throughout the hydrogel. The resulting nano-PANI-based hydrogels exhibited excellent comprehensive performance. The tensile stress–strain curves presented in Figure 6f indicate that the hydrogels possess outstanding stretchability, with a fracture strain exceeding 1000%, while their mechanical strength can be precisely controlled by adjusting the nano-particle content. This combination of high stretchability and tunable mechanics makes them highly suitable for flexible wearable devices. Furthermore, the introduction of glycerol as a binary solvent component endowed the hydrogels with excellent anti-freezing properties. Figure 6g shows that the hydrogels maintained their softness and elasticity even after long-term storage at an extremely low temperature of −28 °C. This remarkable environmental stability greatly expands their application range, highlighting the immense potential of nano-engineered PANI hydrogels in next-generation flexible sensors, wearable electronics operating in harsh conditions, and soft robotic systems.

PPy, as another classic conductive polymer, is also widely applied in constructing conductive hydrogels. However, effectively building continuous and highly efficient PPy conductive networks within the hydrogel matrix while maintaining good mechanical properties remains a challenge. Addressing this issue, He et al. [84] proposed an innovative strategy: utilizing pre-formed polymeric nanofiber networks as templates to guide the in situ polymerization and assembly of pyrrole monomers on their surfaces, thereby forming highly interconnected PPy conductive pathways. Figure 6h schematically illustrates this process, where a nanofiber template formed from aramid nanofibers (ANFs) and PVA guides the polymerization of pyrrole monomers (Py) in the presence of an oxidant (e.g., FeCl_3_) to form a PPy hybrid conductive network. The PPy uniformly coated the pre-constructed ANF-PVA nanofiber framework, creating a homogeneous and highly connected three-dimensional conductive structure. This unique template-assisted assembly significantly enhanced the conductive efficiency of PPy. As shown in Figure 6i, compared to direct PPy polymerization in pure PVA or PAM hydrogels without ANF templates, the PPy composite hydrogels prepared with ANF templates (ANF-PVA-PPy) exhibited electrical conductivities several orders of magnitude higher. At higher PPy content, the conductivity could even reach approximately 80 S cm^−1^, a value significantly surpassing many reported PPy-based conductive hydrogels. Beyond their excellent electrical and mechanical properties, this study also demonstrated the good manufacturability of these conductive nanofiber hydrogels (CNHs) for device fabrication. Through a simple masking technique, the process flow of which is illustrated in Figure 6j, selective in situ polymerization of PPy conductive regions can be achieved on the ANF-PVA hydrogel substrate, enabling the preparation of patterned conductive hydrogel structures. Figure 6k visually presents various patterned CNHs fabricated by this method, such as square arrays, lines, and serpentine structures. This precise patterning capability is crucial for constructing customized flexible electrodes, interconnects, and complex soft bioelectronic devices, paving the way for the practical application of these conductive hydrogels in biomedical sensing, neural interfaces, and tissue engineering.

In summary, carbon materials, liquid metals, and conductive polymer materials serve as the core components of conductive fillers in hydrogels. Through structural design and performance regulation, these materials endow hydrogel systems with diverse functional characteristics: carbon materials, with their high conductivity and mechanical strength, excel in capturing physiological signals and adapting to complex scenarios. Liquid metals overcome durability and conductivity limitations in sensors through their dynamic self-healing and high conductivity advantages [116,117,118]. Conductive polymers optimize interfacial interactions through molecular design, achieving synergistic improvements in biocompatibility and conductivity [119,120,121]. Through different mechanisms and composite strategies, the three components collectively drive advancements in E-skins in terms of sensitivity, stability, and environmental adaptability, providing critical material support and innovative ideas for the deep application of flexible electronics technology in fields such as health monitoring and HMI [122,123].

## 4. Wearable E-Skin Applications

Owing to their unique constellation of properties, including tissue-like softness, remarkable stretchability, inherent biocompatibility, and tunable electrical conductivity, conductive composite hydrogels have emerged as a leading material platform for a new generation of wearable electronic devices [124,125,126]. Their ability to form a stable, conformal, and low-impedance interface with the human skin is the cornerstone of their success, enabling the high-fidelity, non-invasive capture of diverse biophysical and biomechanical signals with minimal motion artifacts [127,128,129]. This section delves into the cutting-edge wearable applications of these advanced hydrogels. Their applications range from electrophysiological signal monitoring, which is revolutionizing the acquisition of critical biomedical data such as ECG and EEG, to the increasingly important field of HMI, where they translate subtle muscle activities and tactile inputs into intuitive commands for controlling virtual and physical systems [130,131,132]. Current hydrogel-based HMI systems can detect muscle contractions with pressure sensitivities ranging from sub-Pascal to several kilopascals and response times typically under 100 milliseconds, though practical implementation requires individual calibration protocols and signal processing algorithms to account for user-specific variations in skin properties and movement patterns [131]. While hydrogel electrodes demonstrate superior performance in reducing skin-electrode impedance and enhancing signal quality compared to conventional dry electrodes, practical limitations remain including signal drift during prolonged wear, sensitivity to environmental moisture variations, and material degradation over time, though they still outperform traditional Ag/AgCl wet electrodes in long-term wearability and user comfort without requiring electrolyte gels. Furthermore, these hydrogels excel in comprehensive body motion monitoring, enabling a wide spectrum of functionalities from decoding complex gestures to multi-degree-of-freedom kinematic analysis, often in synergy with advanced machine learning algorithms [133,134]. These applications collectively underscore the transformative potential of conductive hydrogels in advancing personalized healthcare, intelligent robotics, and seamless bio-integrated interfaces for virtual reality and tactile feedback systems [118,135,136,137].

### 4.1. Electrophysiological Signals Monitoring

Electrophysiological signals, such as EEG, ECG, EMG, and EOG, are crucial indicators of human physiological states and functions, with wide applications in clinical diagnosis, health monitoring, and HMI [138]. Traditional physiological electrodes (e.g., Ag/AgCl gel electrodes), while offering high signal quality, suffer from issues like wearing discomfort, the need for conductive paste, and susceptibility to drying out, making them unsuitable for long-term, dynamic, and imperceptible monitoring [139,140]. In recent years, conductive composite hydrogels have emerged as ideal materials for developing next-generation wearable physiological electrodes due to their excellent biocompatibility, tissue-like softness, tunable conductivity, and potential self-adhesion [141,142,143]. By incorporating conductive fillers (e.g., conductive polymers, carbon materials, MXene) into the hydrogel network, hydrogels can be endowed with good charge transport capabilities, enabling low-impedance, high-fidelity signal coupling with the skin [127,144,145]. This section will focus on recent representative advances in physiological signal monitoring using conductive composite hydrogels [146,147].

**Figure 7:**
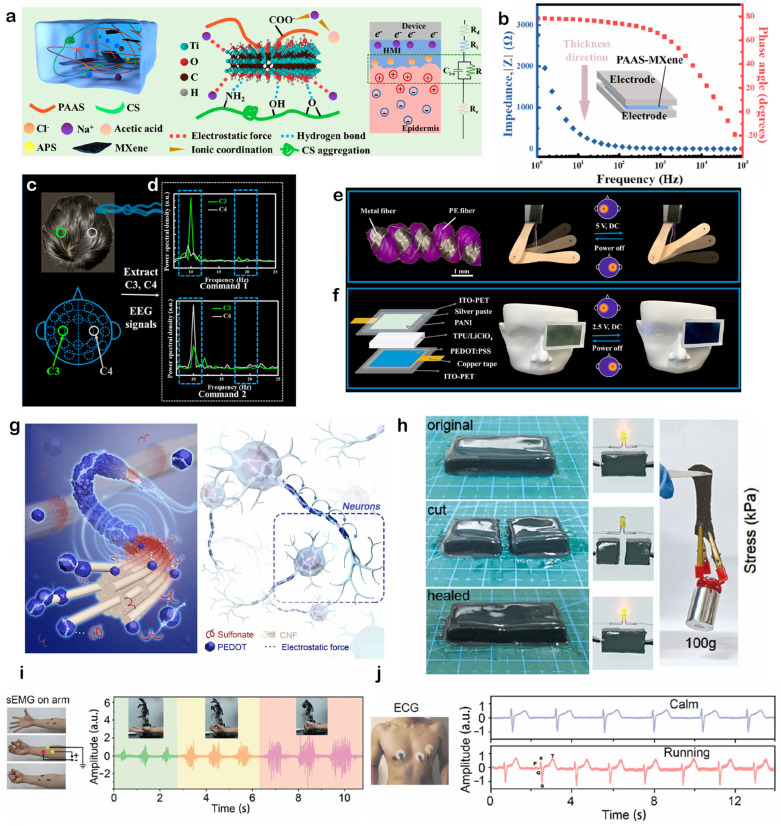
Conductive hydrogels for advanced electrophysiological signal monitoring. (**a**–**f**) An in situ gelling PAAS-MXene hydrogel forms a self-adaptive, low-impedance interface on the scalp, enabling high-fidelity EEG signal acquisition and real-time control of external devices like an artificial muscle and electrochromic glass. Reproduced with permission from Ref. [144], Copyright 2022, American Chemical Society. (**g**–**j**) A biomimetic design inspired by the myelin sheath creates a self-healing hydrogel capable of high-fidelity monitoring of surface electromyography (sEMG) and ECG signals, even during motion. Reproduced with permission from Ref. [148], Copyright 2024, American Chemical Society.

To address the core challenge of traditional electrodes in forming stable, low-impedance interfaces on hairy, uneven scalp surfaces, researchers are actively exploring novel interface materials and strategies. In this context, Luo et al. [144] proposed an innovative solution based on a MXene-enabled self-adaptive hydrogel human–machine interface. The core highlight of this work lies in its “in situ rapid gelation” strategy. A hydrogel precursor solution was designed, composed of sodium polyacrylate, chitosan, and the two-dimensional conductive material MXene, the composition of which is depicted in Figure 7a. This precursor, with its water-like fluidity, could be easily applied to the scalp, bypassing hair obstruction and perfectly filling microscopic skin wrinkles. Under the action of an initiator, this solution rapidly gelled within approximately 5 s, forming a soft, adhesive, and conductive gel electrode that seamlessly conformed to the scalp. This in situ formation capability fundamentally resolved the high contact impedance issues caused by traditional hydrogel electrodes or commercial conductive pastes that fail to fully conform. From an electrical performance perspective, this hydrogel interface exhibited extremely low impedance due to the efficient conductive network constructed by MXene nanosheets and enhanced ion transport. Figure 7b shows that the impedance remains below 50 Ω at physiologically relevant frequencies above 100 Hz, laying the foundation for high-fidelity physiological signal capture. The research team successfully utilized this cap-free method to acquire clear and stable EEG, signals from key brain regions on the scalp, such as C3 and C4. As demonstrated in Figure 7c,d, performing power spectral density analysis on the acquired signals allowed for the effective distinction of characteristic peaks at different frequencies. This enabled the identification of specific user intentions and their decoding into concrete control commands like “Command 1” and “Command 2”. More remarkably, this work demonstrated the immense potential of this self-adaptive hydrogel in active HMI. The acquired and decoded EEG signals were used as real-time control sources to successfully drive external devices. For instance, controlling voltage via EEG signals enabled the contraction and relaxation of artificial muscles, which were twisted from metal and PE fibers. This actuation is visualized in Figure 7e. Simultaneously, the controllable adjustment of electrochromic glass transparency was achieved, a change illustrated in Figure 7f. This clearly demonstrated a complete closed-loop pathway from “brain intention” to “machine execution” and “visual perception change.” The work by Luo et al. not only proposed an ingenious in situ gelation strategy to overcome physical barriers in scalp EEG signal acquisition but also validated its feasibility as a high-performance, imperceptible, and wearable brain–computer interface, providing important material design ideas and application examples for developing next-generation flexible bioelectronic devices for health monitoring and HMI.

An alternative approach to enhancing bio-interfacing performance focuses on the biomimetic design of the hydrogel itself, rather than on the application method. Liu et al. [148] developed a multifunctional composite hydrogel inspired by the myelin sheath structure in the nervous system, which exhibited outstanding performance in acquiring various key electrophysiological signals. The core design concept of this work was to simulate the “saltatory conduction” mechanism of nerve signals between myelin sheaths, a concept schematically illustrated in Figure 7g. This was achieved by constructing a biomimetic PEDOT core-sheath conductive network within the hydrogel matrix to form efficient charge transport pathways. This ingenious biomimetic design not only resulted in excellent electrical properties but also endowed the hydrogel with remarkable mechanical performance and stability. A notable feature is its outstanding self-healing capability: a completely severed hydrogel could rapidly recover its conductive function upon re-contact, sufficient to stably light an LED bulb. This recovery is demonstrated in Figure 7h, indicating excellent durability for wearable device applications. As a high-performance bioelectrode, this hydrogel achieved success in monitoring various electrophysiological signals. In sEMG, monitoring, it could precisely capture signals from arm muscles under different gripping forces, with the signal amplitude directly related to muscle exertion, providing a solid foundation for more refined HMI such as prosthetic control, as shown in the data presented in Figure 7i. In the more demanding application of ECG, monitoring, this hydrogel electrode acquired clear and stable signals with distinct P, Q, R, S, and T waves, regardless of whether the subject was in a calm or running state. This successfully overcame the significant challenge of motion artifacts, a capability highlighted by the recordings in Figure 7j. In summary, this work, through ingenious biomimetic structural design, successfully achieved high-fidelity monitoring of various electrophysiological signals, particularly sEMG and ECG, within a single material system. It provides a valuable materials science example for developing next-generation flexible bioelectronic devices capable of long-term, reliable monitoring during complex daily activities.

### 4.2. Human–Machine Interaction

HMI serves as a bridge connecting humans with the digital world, playing an increasingly important role in virtual reality (VR), augmented reality (AR), gaming and entertainment, intelligent control, and medical rehabilitation [149,150,151,152]. Traditional HMI devices, including keyboards, mice, and game controllers, are often bulky, less portable, and offer limited interaction methods [153,154,155]. E-skins based on flexible conductive materials provide new possibilities for developing more natural, immersive, and portable HMIs [156,157]. Conductive composite hydrogels, owing to their excellent skin conformability, biocompatibility, stretchability, and tunable sensing properties, including piezoresistive, capacitive, and triboelectric mechanisms, demonstrate immense potential in constructing novel wearable HMI sensors [130,150,158,159,160]. These hydrogel sensors can adhere to the human body surface, real-time capturing user movements, gestures, pressure, and other information, converting them into electrical signals to achieve intuitive control of virtual environments or physical devices [133,134,161,162]. This section will introduce some innovative works on HMI sensing applications using conductive composite hydrogels [143,163,164].

**Figure 8:**
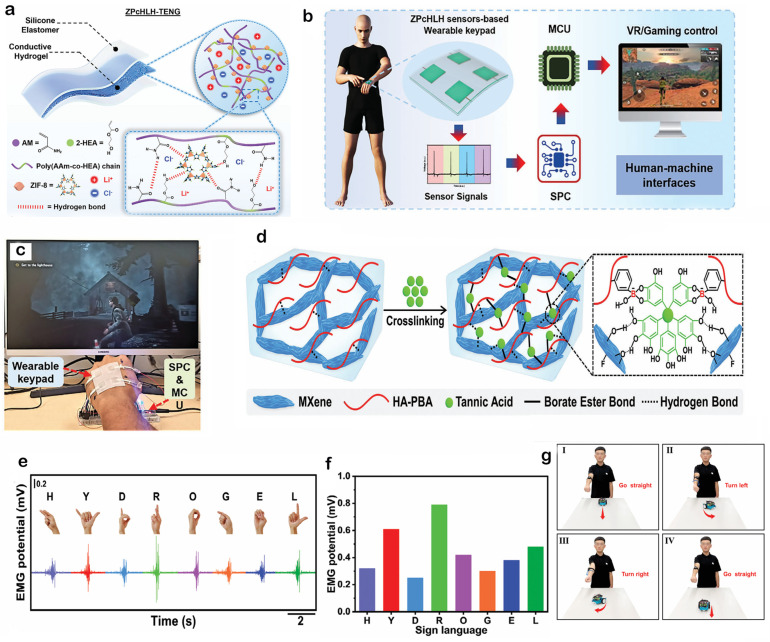
Conductive hydrogels for advanced HMI. (**a**–**c**) A triboelectric nanogenerator (TENG) based on a ZIF-8 reinforced hydrogel is developed into a self-powered wearable keypad for real-time game control. Reproduced with permission from Ref. [35], Copyright 2023, Wiley-VCH. (**d**–**g**) A self-healing MXene-based epidermal sensor records high-fidelity EMG signals for sign language recognition and the wireless control of a smart car, (**g**) demonstrates the car movements in four directions, controlled by corresponding hand gestures: (I) straight, (II) left, (III) right, and (IV) straight Reproduced with permission from Ref. [36], Copyright 2024, Wiley-VCH.

To address the energy supply challenges for wearable electronics, hydrogels with both excellent mechanical properties and energy harvesting capabilities are highly desirable. Rahman et al. [35] developed a highly stretchable and durable conductive hydrogel by incorporating a metal–organic framework, specifically zeolitic imidazolate framework-8 or ZIF-8, as a reinforcing nanofiller into a poly(acrylamide)-co-hydroxyethyl acrylate (PAAm-co-HEA) hydrogel containing a LiCl electrolyte. This nanocomposite hydrogel, hereafter referred to as ZPcHL-hydrogel, forms the core component of a flexible triboelectric nanogenerator, or TENG. The architecture of the resulting ZPcHLH-TENG, featuring the ZPcHL-hydrogel as the central electrode, is schematically illustrated in Figure 8a. The ZIF-8 nanocrystals significantly enhance the hydrogel’s mechanical properties, leading to a 2.7-fold increase in stretchability compared to the pure hydrogel. This material can scavenge biomechanical energy even at sub-zero temperatures and function as a self-powered pressure sensor for human–machine interfaces. The potential of this ZPcHL-hydrogel for advanced human–machine interfaces was further demonstrated through the development of a self-powered wearable keypad system. This system, which utilizes an array of sensors based on the ZPcHL-hydrogel, can determine and recognize human motion states by analyzing differences in output voltage and frequency. The system architecture is schematically illustrated in Figure 8b. The practicality of this wearable interface was successfully validated by using it to control the computer game “Alan Wake,” a demonstration captured in the snapshot in Figure 8c. This highlights the significant potential of ZPcHL-hydrogel-based sensors for applications in VR, robotics control, and electronic skin, paving the way for more intuitive and immersive HMI.

In addition to triboelectric sensing methods based on physical contact like pressure and bending, decoding epidermal electrophysiological signals, especially EMG, offers a new avenue for achieving more refined and intelligent HMI. EMG signals reflect muscle activity, and by precisely capturing and analyzing them, human intentions and actions such as gestures can be identified. However, high-quality EMG signal acquisition places higher demands on electrode interface performance, anti-interference capability, and conformal adhesion to the skin. Furthermore, converting complex biological signals into effective control commands often requires advanced signal processing and machine learning algorithms. Wang et al. [36] have made significant progress in this direction, with their developed multifunctional MXene hydrogel epi- dermal electronics demonstrating immense potential in intelligent HMI. They fabricated MXene hydrogels (MXene/HA-PBA/TA) by cleverly assembling a MXene (Ti_3_C_2_T_x_) nanosheet network with a self-healing polymer hydrogel network composed of the natural polyphenol TA and phenylboronic acid-grafted hyaluronic acid (HA-PBA). To obtain unexfoliated Ti_3_C_2_T_x_ with an accordion-like microstructure, the Ti_3_AlC_2_ MAX phase was etched with HCl/LiF to remove the Al layers, a process illustrated in Figure 8d. This facilitated the convenient fabrication of self-adhesive, UV-protective, and antibacterial MXene hydrogel-based epidermal sensors. By combining sign language gesture recognition based on EMG signals collected by these sensors with advanced machine learning algorithms, high-precision gesture classification can be achieved. As shown in Figure 8e, EMG signals of eight different sign language gestures can be monitored by the MXene/HA-PBA/TA hydrogel-based sensors attached to a volunteer’s right arm. The signals exhibit distinguishable amplitudes, which are clearly depicted in the recordings in Figure 8f. Furthermore, intelligent human–machine interface control systems based on collected EMG signals are attracting increasing attention. Figure 8g presents a flowchart of an intelligent wireless car control system, which includes EMG signal acquisition, data processing, wireless transmission, and car movement control. By wearing an armband integrated with the MXene/HA-PBA/TA sensors, real-time control of a synchronous intelligent wireless car was achieved. This work successfully demonstrates a complete pipeline from high-fidelity biological signal acquisition to intelligent decoding and real-time machine control, showcasing the power of combining advanced materials with machine learning for sophisticated HMI.

### 4.3. Body Motion Monitoring

Human motion monitoring is one of the most important and widespread application areas for wearable electronic devices [12,165,166]. By attaching flexible sensors to the human skin or integrating them into clothing, a series of complex dynamic information, ranging from subtle muscle tremors and laryngeal vibrations caused by vocalization to large-scale joint bending and walking gaits, can be captured in real-time and non-invasively [5]. This information holds immeasurable value in fields such as sports science, rehabilitation medicine, HMI, and VR [167,168,169]. However, achieving high-precision, comprehensive motion monitoring places extremely high demands on conductive hydrogel sensors, requiring them to possess not only high sensitivity, a wide detection range, and rapid response, but also excellent mechanical robustness such as high stretchability and fatigue resistance. Furthermore, they must be capable of handling multi-degree-of-freedom, multi-modal complex movements and effectively parsing meaningful instructions [170,171,172].

**Figure 9:**
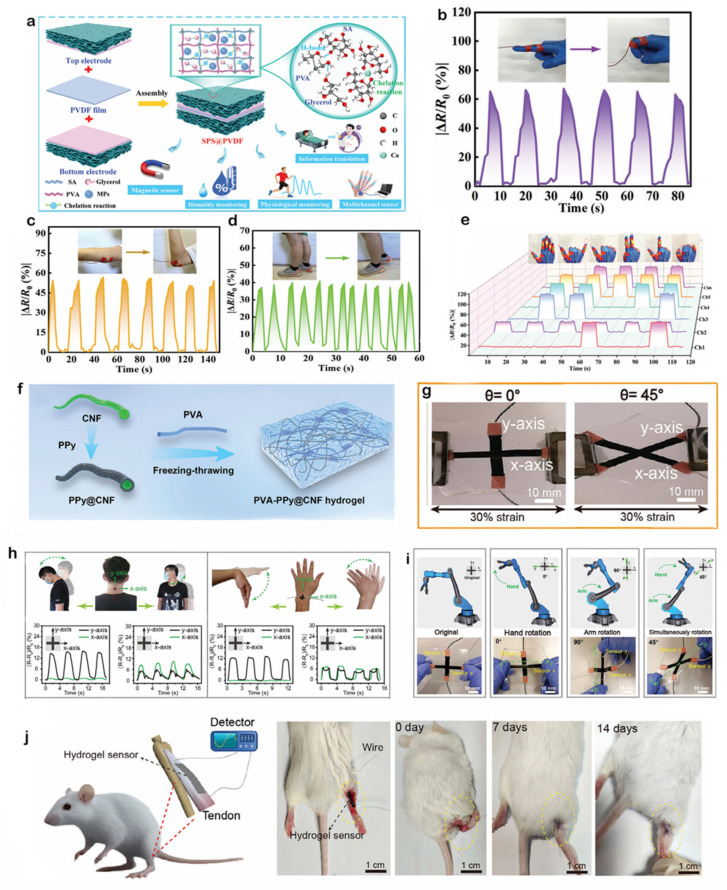
Conductive hydrogels for advanced body motion monitoring. (**a**–**e**) A sensor array based on a hydrogel sponge is combined with deep learning algorithms to achieve high-accuracy sign language recognition for wireless car control. Reproduced with permission from Ref. [37], Copyright 2024, Wiley-VCH. (**f**–**j**) An anisotropic hydrogel, inspired by muscle fibers, enables multidirectional strain sensing for complex motion decoding, robotic arm control, and in vivo monitoring. In (**j**), dashed circles indicate the implantation site of the hydrogel sensor at different time points post-implantation. Reproduced with permission from Ref. [173], Copyright 2024, Wiley-VCH.

To achieve precise decoding of complex human movements, relying solely on individual sensors is far from sufficient. The current mainstream trend involves developing high-performance sensor arrays combined with intelligent algorithms for pattern recognition. In this regard, Fu et al. [37] developed a multimodal sensor based on a sodium alginate/polyvinyl alcohol (SA/PVA) hydrogel sponge, which successfully achieved complex sign language recognition by integrating deep learning algorithms. The ingenuity of this work lies in the construction of a multifunctional composite structure. As illustrated in Figure 9a, they hierarchically assembled conductive hydrogels with porous sponge structures (SPSs) and PVDF films, fabricating a lightweight, flexible, multilayer sensor (SPS@PVDF). This sponge-like porous structure not only endowed the sensor with excellent flexibility and compressibility but also provided a large specific surface area for conductive pathways and sensing interfaces. Basic performance tests indicated that the sensor could reliably monitor various fundamental human movements. For instance, when attached to finger joints, elbow joints, or the sole of the foot, it could clearly and stably capture resistance change signals generated by joint bending and walking motions. These capabilities are demonstrated in Figure 9b, Figure 9c, and Figure 9d, respectively, showcasing its effectiveness as a basic motion monitoring unit. This work further integrates sensor arrays with deep learning, achieving a leap from simple motion perception to complex intention recognition. Multiple sensor units were integrated into a glove, constructing a sensor array capable of synchronously capturing different finger bending and collaborative states. Figure 9e shows that when the wearer performed different gestures like “accelerate”, “decelerate”, “turn left”, and “turn right”, the six channels on the glove generated unique and repeatable combined signal patterns. These high-dimensional temporal signals were extremely difficult to distinguish using traditional methods, but by introducing a deep learning model combining a Convolutional Neural Network (CNN) and a Gated Recurrent Unit (GRU), the system could classify and recognize these complex gestures with an accuracy of up to 99.17%. While this high accuracy was achieved on a laboratory dataset with controlled conditions, practical deployment would require extensive validation across diverse users and environments, along with addressing sensor durability challenges such as signal degradation after repeated cycling and moisture-induced baseline drift during extended wear periods. Ultimately, the recognized gesture commands were used to wirelessly control a toy car, fully demonstrating a closed-loop HMI from human motion acquisition and intelligent decoding to machine control. This work not only provided a high-performance hydrogel sponge sensing material but also offered a highly valuable paradigm and systematic solution for using multi-channel sensing data and artificial intelligence techniques to address the challenge of complex motion recognition, such as in sign language.

An alternative strategy for complex motion recognition shifts the focus from multi-sensor fusion to the constitutive design of the material itself, aiming to achieve multi-dimensional, anisotropic movement perception at the single-sensor level. This bottom-up approach seeks to simplify the complexity of sensing systems and data processing by endowing materials with inherent directional sensitivity. In this regard, Lin et al. [173] developed a conductive hydrogel with significant anisotropy for multidirectional strain sensing, inspired by the highly ordered structure of human muscle fibers. Their core idea was to construct oriented conductive networks within the material through structural biomimicry, thereby achieving direction-selective sensing responses. This work first blended conductive polypyrrole-modified cellulose nanofibers (PPy@CNF) with PVA to form an isotropic hydrogel, as shown in the initial state in Figure 9f. The crucial step involved mechanically pre-stretching the hydrogel to orient the polymer chains and conductive networks along the stretching direction, followed by post-crosslinking with TA to permanently fix this ordered anisotropic structure, successfully mimicking the arrangement of muscle fibers. This structural anisotropy directly translated into electrical anisotropy. When tensile strain was applied parallel to the fiber alignment, the conductive network was effectively disrupted, and resistance significantly increased, exhibiting high sensitivity. Conversely, when strain was applied perpendicular to the fiber direction, the conductive network was less affected, and the resistance change was minimal. Leveraging this characteristic, a multidimensional strain sensor was ingeniously constructed by orthogonally stacking two anisotropic hydrogel strips, the structure of which is depicted in Figure 9g. This direction-resolving capability was validated across multiple joints. For instance, when used to monitor neck and wrist movements, as demonstrated in Figure 9h, the sensor could clearly distinguish between single-directional actions like nodding or wrist flexion and compound-directional actions such as head rotation. The distinct signal responses from the two channels enabled precise resolution of multi-degree -of-freedom movements. This capability was further utilized to construct a more intuitive HMI system. Figure 9i shows that researchers mapped the signals from the sensor’s two orthogonal channels to the Hand and Arm joints of a virtual robotic arm. When stretching was applied along only one direction, only the corresponding joint underwent independent rotation. When stretching was applied at a 45° angle, both joints moved simultaneously and coordinately. This intuitively demonstrated that the sensor, solely by virtue of the material’s inherent anisotropy, achieved decoupled control of a multi-degree-of-freedom system, offering a new avenue for developing intelligent interactive devices with lower power consumption and less computational dependence. Another major highlight of this work was its demonstration of potential as an implantable bioelectronic device. As illustrated in Figure 9j, the hydrogel sensor was successfully implanted into the Achilles tendon of mice, owing to its excellent biocompatibility and anti-biofouling properties. Over a 14-day period, it consistently and reliably monitored tendon movements, demonstrating immense application prospects in tissue repair and long-term in vivo health monitoring.

In summary, this section has systematically demonstrated the extensive applications of conductive composite hydrogels in key areas such as electrophysiological signal monitoring, HMI, and body motion monitoring through a series of cutting-edge examples [114,174,175]. From utilizing in situ gelation strategies to overcome interfacial challenges, to employing biomimetic structural designs inspired by biological systems like myelin sheaths and muscle fibers, and integrating multi-sensor arrays with deep learning algorithms, these studies collectively prove that conductive composite hydrogels are not merely simple sensing elements, but rather core functional materials for building next-generation intelligent, imperceptible, and closed-loop wearable systems [176,177]. While these achievements are impressive, overcoming challenges related to long-term stability and scalable manufacturing is crucial for their widespread commercial and clinical translation [178].

## 5. Conclusions and Future Perspectives

In summary, this review has provided a comprehensive overview of recent advancements in conductive composite hydrogels engineered for wearable sensing and electrophysiological monitoring. The strategic integration of functional nanomaterials, including carbon-based materials, metallic nanomaterials, and two-dimensional transition metal compounds, with biocompatible polymer matrices derived from both synthetic and natural sources has yielded a new class of materials. These composites exhibit a unique synergy of properties, including robust electrical conductivity, superior mechanical characteristics such as high stretchability and tunable modulus, and excellent biocompatibility. These hydrogels function as enabling platforms for next-generation wearable E-skin systems, facilitating high-fidelity, long-term monitoring of critical bio-signals, such as ECG, EMG, and EEG, and supporting sophisticated applications in HMI and biomechanical analysis. The demonstrated capabilities confirm that conductive composite hydrogels represent a promising technology for the future of intelligent, conformal, and closed-loop wearable electronic skins.

Despite evaluation revealing that while these materials show promising laboratory performance, they still face significant gaps compared to established commercial sensors in terms of long-term stability, batch-to-batch reproducibility, and manufacturing costs, indicating that substantial technical challenges remain before widespread clinical adoption becomes feasible. The foremost of these is ensuring long-term operational stability, issues related to dehydration, ionic leakage, and performance degradation under variable physiological and environmental conditions remain critical challenges to overcome. Meanwhile, while initial biocompatibility is often established, the long-term in vivo safety, including potential immunogenicity and the effects of degradation byproducts, requires more rigorous and systematic investigation, particularly for implantable device paradigms. Furthermore, enhancing signal fidelity through reduced skin-electrode impedance and advanced signal processing algorithms to mitigate motion artifacts is crucial. Also, the development of cost-effective, scalable, and reproducible manufacturing techniques is desirable to bridge the gap between laboratory-scale synthesis and industrial production. From a preparation perspective, current challenges include maintaining uniform pore structure, consistent filler dispersion, and reproducible crosslinking density across large-scale production, which directly impact the electrical and mechanical properties of the final devices. Moreover, the integration of artificial intelligence and machine learning algorithms presents unprecedented opportunities for real-time signal interpretation and personalized health monitoring through pattern recognition of complex physiological data. Advanced manufacturing approaches, particularly 3D/4D printing technologies, offer promising pathways for scalable fabrication of hierarchically structured hydrogels with programmable stimuli-responsive properties, enabling customized E-skin devices tailored to individual patient needs. Future research will likely focus on creating multifunctional hydrogel platforms capable of multi-modal sensing, as well as advancing “theranostic” systems that integrate diagnostic monitoring with therapeutic interventions. It is worth noting that incorporating multiple functionalities inevitably introduces trade-offs. For instance, adding therapeutic drug-delivery capabilities may compromise mechanical integrity or electrical conductivity, thus necessitating careful optimization of material compositions and structural designs to balance competing performance requirements. The continued interdisciplinary collaboration between materials science, electronic engineering, and computational science holds the promise of advancing these advanced hydrogels to revolutionize personalized healthcare and HMI technology.

## Figures and Tables

**Table 1:** Performance comparison of commonly used conductive fillers in composite hydrogels.

Polymer Matrix	Conductive Fillers	Tensile Strain %	Breaking Strength	Electrical Conductivity	Young’s Modulus	Gauge Factor (GF)	Adhesion Strength	Toughness	Reference
XSBR/SS	CNTs	217	12.58 MPa	0.071 S/m	1.19 MPa	25.98	/	6.23 MJ/m^3^	[78]
LG/TA	Graphene	1860	200 kPa	28 S/m	/	4.61–346	51.3 kPa	/	[73]
PNIPAM	CF	1200	3.0 ± 0.3 MPa	670 S/m	74 ± 7.0 MPa	6–647	/	0.9 MJ/m^3^	[74]
PAA/TA	EGaIn	2000	/	28.3 mS/m	6.24 kPa	1.878	0.96 MPa	/	[79]
CNCs/PAA	EGaIn	2000	70 kPa	3.8 S/m	49–98 kPa	/	/	1.8 MJ/m^3^	[80]
MXene/PNIPAM	EGaIn	610	0.05 MPa	/	/	3.25–8.92	1–4 kPa	/	[81]
P (PEG-co-AA)	PANI	580–1030	25 kPa	74.32 mS/cm	6 kPa	1.46–2.43	/	75–110 kJ/m^3^	[82]
PU	PEDOT:PSS	400	/	11 S/cm	1 MPa	/	/	>3300 J/m^2^	[83]
PVA/ANFs	PPy	36	9.4 MPa	80 S/cm	25–35 MPa	0.2–0.7	/	2000–2500 J/m^2^	[84]

## Data Availability

No new data were created in this work.
